# Ehrlichiosis: A Unique Presentation of Fatal Sepsis in an Immunocompetent Adult

**DOI:** 10.7759/cureus.53932

**Published:** 2024-02-09

**Authors:** Lauren N Mazin, Christopher J Peterson, Christi A Stewart

**Affiliations:** 1 Internal Medicine, Carilion Clinic, Roanoke, USA; 2 Palliative Care, Carilion Clinic, Roanoke, USA

**Keywords:** tick-borne disease risk factors, fatal ehrlichiosis, amblyomma americanum, tick-borne disease, ehrlichiosis, ehrlichia chaffeensis, lone star tick, tick-borne illness

## Abstract

Ehrlichiosis is a vector-borne illness transmitted by the lone star tick (*Amblyomma americanum*). Most patients have risk factors for tick exposure, such as hobbies or careers involving hunting, camping, and hiking. This case exposes a rare case of severe ehrlichiosis, ultimately resulting in fatal sepsis, in an elderly patient without any reported tick bites or exposures. This patient had a history of cognitive impairment, which was confounded by acute encephalopathy at presentation. Unfortunately, this hindered his ability to report any known tick exposures, which posed a challenge in the diagnosis and ultimately delayed treatment as there were no clear findings of a tick bite or known exposures.

## Introduction

Human monocytic ehrlichiosis (HME) is a tick-borne illness caused by *Ehrlichia chaffeensis*, a gram-negative, obligate intracellular bacteria first identified in 1986 [1]. HME typically presents with nonspecific symptoms of fever, malaise, headache, and myalgia, with 20% of patients having confusion. Laboratory abnormalities include thrombocytopenia, leukopenia, and elevated liver enzymes. Although a rash is often associated with tick-borne illnesses, only a minority of adult patients with HME have a maculopapular rash [2]. Present in most cases of ehrlichiosis is a known tick bite or exposure, or activities that place one in proximity to ticks, such as certain forms of outdoor recreation. Nevertheless, the absence of a tick exposure or at-risk activities does not preclude ehrlichiosis. In fact, the absence of tick bites may actually increase the risk of serious illness and poor outcomes due to delay in diagnosis and treatment. In these cases, diagnosis can be especially challenging. Here, we present a challenging case of ehrlichiosis sepsis/HME in an immunocompetent patient with no confirmed tick bite marks. Due to the patient's cognitive impairment, it was not possible to interview him directly regarding whether or not he had been bitten by or exposed to ticks.

## Case presentation

An 81-year-old male in the South Atlantic United States with a past medical history pertinent for essential hypertension, hyperlipidemia, paroxysmal atrial fibrillation, previous cerebrovascular accident, cognitive changes following a motor vehicle accident, and depression presented with a two-day history of confusion, lethargy, and decreased appetite in the summer. Although the patient did have dementia at baseline, his family had noted worsening weakness and lethargy prior to presentation. His family also reported that the patient spent most of his time indoors. He enjoyed hunting; however, he had not been hunting in about 10 years. He did not spend time in the woods, hike, bike, camp, or perform other outdoor activities. He did not have any pets at home and did not have any recent travel.

On the morning of presentation, the patient woke up unable to recognize his family member and unable to walk without assistance. Initially, the patient was afebrile and mildly hypertensive, with normal heart rate and oxygen saturation. Initial laboratory results were notable for leukopenia, thrombocytopenia, bilirubinemia, mild transaminitis, elevated C-reactive protein (CRP), elevated procalcitonin, and elevated alcohol level (Table 1). The urine drug screen was negative. Acute encephalopathy was thought to be secondary to urinary tract infection; however, urinalysis returned only with sterile pyuria. Magnetic resonance imaging of the brain was obtained and was negative for any acute abnormalities. A chest X-ray did not demonstrate any definite infiltrates. On day 2 of the hospital course, the patient became febrile with a temperature of up to 103.2°F. Repeat laboratory results at that time revealed worsening leukopenia, worsening thrombocytopenia, and rising procalcitonin (Table 1). The patient was started on broad-spectrum antibiotics with vancomycin and cefepime. The etiology of sepsis remained unclear as urinary tract infection, acute intracranial abnormalities, and pulmonary pathology had been ruled out. Additionally, skin survey did not reveal any ulcerations or injuries, and blood cultures were negative. On day 3, doxycycline was added to the therapy regimen given concern for tick-borne illness in the setting of leukopenia, thrombocytopenia, and persistent fevers. The patient became increasingly unstable as his hospital course continued. He developed lactic acidosis, worsening leukopenia, thrombocytopenia, transaminitis, persistent fevers, atrial fibrillation with rapid ventricular response requiring a diltiazem drip, and pulmonary edema and respiratory distress requiring noninvasive ventilation. Tick-borne panel returned on day 6 of the hospital course positive for *Ehrlichia chaffeensis* (Table 2). Blood counts were thought to be improving; however, the patient eventually developed leukocytosis on day 8 of the hospital course. Unfortunately, the patient's clinical status continued to deteriorate. He remained encephalopathic and minimally responsive with worsening respiratory distress. The patient's spouse ultimately decided to pursue comfort measures. The patient was transferred to the palliative care unit where he passed peacefully on day 12.

**Table 1:** Pertinent laboratory findings from the hospital course WBC: white blood cell count, ALP: alkaline phosphatase, AST: aspartate aminotransferase, ALT: alanine transaminase, CRP: C-reactive protein *Leukopenia nadir occurred on day 3. **Thrombocytopenia nadir occurred on day 4 (not included in the table) with a platelet count of 24 K/uL.

Component	Admission/day 1	Day 2	Day 3	Day 8	Reference range
WBC	3.1 K/uL	1.8 K/uL	1.4 K/uL*	11.1 K/uL	4.0-10.5 K/uL
Hemoglobin	14.1 g/dL	13.3 g/dL	11.9 g/dL	11.4 g/dL	13.0-16.0 g/dL
Platelet count	114 K/uL	60 K/uL	28 K/uL**	104 K/uL	130-400 K/uL
Total bilirubin	1.9 mg/dL	1.6 mg/dL	1.3 mg/dL	1.3 mg/dL	0.2-1.1 mg/dL
ALP	82 U/L	67 U/L	100 U/L	90 U/L	46-116 U/L
AST	39 U/L	56 U/L	138 U/L	424 U/L	15-37 U/L
ALT	28 U/L	33 U/L	58 U/L	398 U/L	10-49 U/L
CRP	1.8 mg/dL	-	-	-	<1.0 mg/dL
Sedimentation rate	8 mm/hour	-	-	-	0-20 mm/hour
Lactic acid	-	1.0 mmol/L	3.8 mmol/L	1.2 mmol/L	0.4-2.2 mmol/L
Procalcitonin	0.19 ng/mL	0.26 ng/mL	-	-	<0.05 ng/mL
Blood alcohol level	0.004% wt/vol	-	-	-	<0.003% wt/vol
Ammonia	20 umol/L	-	-	-	11-32 umol/L

**Table 2:** Tick-borne illness acute molecular panel DNA: deoxyribonucleic acid, PCR: polymerase chain reaction

Component (DNA, PCR)	Detection
Anaplasma phagocytophilum	Not detected
Babesia microti	Not detected
Borrelia miyamotoi	Not detected
Ehrlichia chaffeensis	Detected
*Borrelia* species	Not detected

## Discussion

Ehrlichiosis is an emerging infectious disease driven by vector exposure. *Ehrlichia chaffeensis* is endemic to the southeastern and south-central United States and is transmitted predominantly by arthropod vectors, with *E. chaffeensis* being transmitted by the *Amblyomma americanum* tick [1]. Exposure typically occurs from May to August, which coincides with the peak feeding activity of *A. americanum* [1]. Most patients have known or suspected exposure to ticks. Indeed, approximately 70% of patients report a tick bite within 30 days of presentation [3]. However, as presented here, there are important exceptions to ehrlichiosis with known or obvious tick exposure. In several instances of critical illness, patients were found unconscious in the woods prior to hospitalization, suggesting a high likelihood of tick exposure [4]. Furthermore, although rare, transmission without tick exposure may occur in individuals who come into direct contact with infected blood. For example, *Ehrlichia* are stable in refrigerated blood products for up to 11 days, and multiple cases of ehrlichiosis have been reported due to blood transfusion [5,6]. Outdoor recreation is also a risk factor for infection [7]. Butchers and hunters may come into contact with blood from infected animal carcasses, although this mode of transmission is debated [3,8,9]. Males have been shown to have a higher risk of infection, presumably due to higher rates of outdoor recreation such as hunting that could lead to animal exposure and thus tick exposure [1]. Other atypical forms of transmission include donor-derived solid organ transplantation and perinatal transmission [10-12]. As animals can harbor ticks, there has been some concern that pet ownership may increase the risk for ehrlichiosis infections, although no association has been proven [13].

This case is a rare reported instance of severe ehrlichiosis in a patient without a clear risk of transmission, such as via tick bites. Although reports of ehrlichiosis without tick exposure have been reported in retrospective studies, there are few individual reports about patients infected without evidence of tick exposure or other risk factors, such as outdoor activities. A literature search demonstrated only one case with similarly unknown transmission, a fatal case of ehrlichiosis in a 34-year-old immunocompetent female in Mexico without evidence of tick bites [14]. Of note, one study determined that lack of tick bites was actually correlated with worse outcomes, initially presumed to be differences in time to initiation to doxycycline, although this relationship still held when doxycycline treatment was controlled for. The authors suspected that the inability to communicate tick exposure in critically ill patients may contribute to worse outcomes in these patients [15].

Physicians should not consider the absence of tick bites sufficient to rule out the possibility of ehrlichiosis in endemic areas, particularly in patients who are not able to provide a history. Given the low likelihood of atypical forms of transmission, it is likely that *E. chaffeensis* was transmitted via tick bite in this case, despite the lack of bites on examination. Patients with dementia could theoretically be at higher risk for ehrlichiosis given the potential for reduced ability to report tick exposure, reduced personal hygiene, and lower vigilance to surveil and remove ticks. Additionally, with an incubation period of 5-14 days, symptoms may not be present in the immediate days following tick exposure and could occur after a bite has healed [16]. PCR and ELISA detection has provided a rapid and sensitive means for diagnosing ehrlichiosis, as well as screening for other tick-borne diseases, and should be considered if tick-borne illness remains uncertain. Doxycycline remains the standard treatment for ehrlichiosis, as well as many other rickettsial illnesses [1]. Early administration of doxycycline can be crucial in patients with suspicion of tick-borne illnesses due to the risk of rapid, severe progression without treatment [2].

## Conclusions

In summary, while most cases of ehrlichiosis are precipitated by a known tick bite or history of outdoor recreation activities, the absence of these does not preclude ehrlichiosis. Indeed, the absence of tick bites may actually give rise to poor outcomes due to delay in diagnosis. This case highlights the potential for ehrlichiosis in patients without evidence of tick bites or known tick exposure. Patients unable to provide a thorough history, such as those with dementia, may be at even higher risk. In conclusion, patients with unexplained sepsis and thrombocytopenia, with residence in endemic areas, may benefit from empiric doxycycline coverage due to the consequences of delayed treatment of ehrlichiosis.
